# Brimonidine tartrate 0.15% drops to reduce low post-LASIK residual myopia: A retrospective study

**DOI:** 10.1371/journal.pone.0329364

**Published:** 2025-07-30

**Authors:** Nir Gomel, Asaf Achiron, Nadav Shemesh, Elad Eilon, Tal Yahalomi, Dana Barequet, Nadav Levinger, Dafi Porat, Shmuel Levinger, Ami Hirsch, Nir Sorkin, Eliya Levinger

**Affiliations:** 1 Department of Ophthalmology, Tel Aviv Sourasky Medical Center and Sackler School of Medicine, Tel Aviv University, Tel Aviv, Israel; 2 Enaim Refractive Surgery Centers, Tel Aviv, Israel; 3 The Faculty of Medicine, The Hebrew University of Jerusalem, Jerusalem, Israel; 4 Department of Ophthalmology, Samson Assuta Ashdod Hospital and the faculty of Health Sciences, Ben-Gurion University of the Negev, Ashdod, Israel; 5 Department of Ophthalmology, Hadassah Medical Center, Jerusalem, Israel; 6 Rabin Medical Center, Tikva, Israel; 7 Kittner Skin Cancer Screening & Research Institute, Sheba Medical Center, Tel Aviv, Israel; University at Buffalo Jacobs School of Medicine and Biomedical Sciences: University at Buffalo School of Medicine and Biomedical Sciences, UNITED STATES OF AMERICA

## Abstract

**Purpose:**

Residual myopia following Laser-assisted in situ keratomileusis (LASIK) surgery poses a significant concern, with existing literature extensively detailing the use of timolol for treatment. This study aims to assess prediction factors for brimonidine tartrate 0.15% (Alphagan-P) response on post-LASIK residual myopic refraction reduction.

**Methods:**

The study included consecutive patients who received Alphagan-P during their follow-up for post-LASIK residual myopia.

**Results:**

We included 61 patients (55% male) with a mean age of 35.18 ± 10.13 Alphagan-P Treatment started at a mean of 4.94 ± 5.64 months after surgery for residual myopia of −0.53 ± 0.71D. Comparison analysis of patients who responded (n = 32, 51.6%) to patients who did not (n = 30, 48.4%) shows that responders were older (38.1 ± 9.1 vs. 32.3 ± 10.3, p = 0.03), had higher baseline myopic Spherical equivalent (SE, −0.82 ± 0.65 vs. −0.26 ± 0.66 p < 0.01), and lower uncorrected visual acuity (Uncorrected visual acuity [UCVA], 0.14 ± 0.2 vs. −0.003 ± 0.12 p = 0.01). Multiple Binary logistic regression confirmed these predictors for response (UCVA (OR=70.6, *P* = .006), larger SE (OR=3.8, *P* = .004,) and older age (OR=1.06, *P* = .03)).

**Conclusions:**

Alphagan-P can reduce up to 0.5D of post-LASIK residual myopia in roughly 50% of subjects. This treatment might be recommended to fine-tune outcomes for low residual myopia.

## Introduction

Myopia is the most common refractive error, with an expected increase in global prevalence from 23% in 2000 to 50% in 2050 (5 billion people) [1]. This estimation may be even larger as myopia among schoolchildren significantly increased during the COVID-19 outbreak due to limited outdoor play and increased digital learning [2].

**Fig 1:**
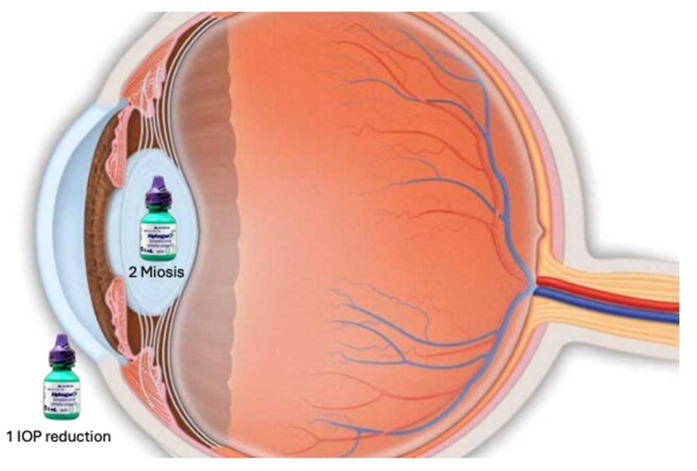
(1) The combination treatment effect of IOP-10 lowering with the rearward displacement of the cornea and flattening of its curvature. (2) The pinhole effect through miosis promotes depth of focus. This results in halo reductions, improved night vision and contrast, and reduced discomfort and subconjunctival hemorrhage.

Refractive surgery is a widely performed procedure with high patient satisfaction. Technological improvements in corneal reshaping excimer laser systems and eye-tracking software have yielded unprecedented results, with more than 200 million surgeries performed worldwide [3]. Laser in situ keratomileuses (LASIK) are safe, effective, and efficient for correcting myopia. It has become a valid treatment option for patients with high visual demands seeking spectacle and contact lens independence [4]. While refractive surgery alters and reshapes the cornea to modify its refractive power, it may not stop residual myopia or progression, which may be caused by an accuracy mistake of the laser used during the procedure or by changes in the cornea with time [5]. The retreatment rate after refractive surgery has been reported to be between 0.48–16%, depending on clinical factors such as age, amount of sphere and astigmatism, ablation depth, keratometric power, and surgeon experience [6]. However, in cases with lower residual myopia, where retreatment of ablation may not be a valid option (for example, up to −0.5D, intraocular pressure (IOP) reduction with beta-blockers agents resulted in mild refraction change by reducing the posterior corneal anterior bowing, Table 1, Fig 1 [5,7–10]. To our knowledge, all previously published data studied the effect of timolol eye drops exclusively.

**Table 1:** Previous studies compared intraocular pressure eye drops on residual myopia following refractive surgery.

Author	Country	Year	Type of eye drops	No. of eyes	Follow-up time (months)	Results
El-Awady, Ghanem & Gad	Egypt	2010	Timolol 0.1% gel	95	12	Timolol, 0.1% gel, effectively reduced and improved residual myopia.
Qi et al.	China	2017	Timolol 0.5 eye drops	62	5	Timolol was effective for 0.5-D, or a more significant myopic shift was detected after LASIK. Residual myopia recurred after the cessation of Timolol treatment.
Shojaei et al.	Iran	2012	Timolol 0.5 eye drops	124	6	Timolol application effectively treats residual myopia after LASIK compared with the control group.
Singh et al.	India	2020	Timolol 0.5 eye drops	29	9	Timolol is effective for the treatment of residual myopia post-LASIK. The most probable mechanism is a reversal of the anterior bowing of the cornea in response to intraocular pressure changes.

LASIK laser-assisted in-situ keratomileusis.

In our center, we have used Brimonidine tartrate 0.15% ophthalmic solution (Alphagan-P) drops for pupil constriction, especially in mesopic conditions that may reduce night glare and halos [11–13]. Alphagan-P is a selective alpha2-adrenergic receptor agonist, and it also reduces IOP within 1 hour of dosing, with a peak effect occurring at 2–3 hours [14]. This study aims to evaluate our clinical experience with Alphagan-P on patients with residual myopia following LASIK treatment.

## Materials and methods

All data for the study were collected and analyzed following the policies and procedures of the Institutional Review Board of the Tel Aviv Medical Center and the tenets outlined in the Declaration of Helsinki (protocol number 0689 17-TLV). The ethical committee waived informed consent for this retrospective data analysis study.

### Study participants

This retrospective case-series study included consecutive subjects who underwent LASIK at the Enaim Refractive Surgery Center, Jerusalem, Israel, by one surgeon (SL and had a history of postoperative use of Alphagan-P drops once a day. Data were obtained through the computerized database registry, including patient demographic and clinical data variables, archived by electronic record-keeping software. Records are updated by the center’s staff prospectively during each patient’s visit. Inclusion criteria were patients with manifest refraction of spherical equivalent greater than −0.25D and decreased distant visual acuity. We excluded patients receiving Alphagan-P drops for night-related symptoms as halos and included only those who had residual myopia.

### Data collection

The medical files of all eligible subjects were reviewed, and the following demographic and pre-operative information was extracted: age, gender, pre-operative refractive error (sphere, spherical equivalence (SE), and cylinder), uncorrected distance visual acuity (UCVA), and best corrected distance visual acuity (BCVA). The following intraoperative information was extracted: the involved eye (right or left), treatment zone, ablation depth, and complications during the procedure. Post-operative information, both pre and post-Alphagan-P treatment, included refractive error, UCVA, and BCVA. All parameters were normally distributed. Refractive exams were conducted by experienced professional opticians and were taken under the same conditions (light, the distance between optician and patient. We included the right eye to reduce inter-eye correlation bias in cases where both eyes were treated [15]. Non-responders were defined by having a difference in SE < 0 (myopic shift) between baseline and post-treatment with Alphagan-P. Responders were defined as subjects who had a change in SE > 0D following treatment. Our steroid tapering regime included starting Dexamethasone 0.1% 6 times a day for the first week, 4 times a day for the second week and then stopped.

All data were accessed in 01-June 2024

### Statistical analysis

Statistical analysis was performed using SPSS software (version 23; IBM Corporation, Armonk, NY) and MedCalc software (version 12.5; MedCalc Software, Inc., Mariakerke, Belgium). We used the paired and independent samples T-test to compare related and unrelated variables. We conducted Fisher’s exact test for categorical variables. A multiple logistic regression analysis was conducted to predict response to treatment. A P-value of less than 0.05 on a two-sided test was considered statistically significant. Due to the non-normal distribution of spherical equivalent values in the non-responder group, we have presented the data as mean ± SD for consistency, but acknowledge that median (range) may better reflect the underlying distribution. No patients were included with SE > –0.25D based on the most recent preoperative measurement used for eligibility.

## Case history

Sixty-one eyes of 61 patients (55.7% male) with a mean age of 35.18 ± 10.13 years were included in this study. Refraction and visual acuity data are presented in Table 2. Alphagan-P Treatment started at a mean of 4.94 ± 5.64 months after surgery for residual myopia of −0.53 ± 0.71D and continued for mean of 2.9 ± 3.4 months. A comparative analysis of patients who responded (n = 30, 49.2%) to those who did not (n = 31, 50.8%) is presented in Table 3. Responders were older (38.1 ± 9.1 vs. 32.3 ± 10.3 years, p = 0.03), had higher baseline SE (0.82 ± 0.65 vs. −0.26 ± 0.66 p < 0.01), and lower uncorrected visual acuity (UCVA, 0.14 ± 0.2 vs. −0.003 ± 0.12 logMAR, p = 0.01).

**Table 2:** Refraction and visual acuity data pre-operatively, before, and after Alphagan-P treatment.

	Sphere	Cylinder	Spherical equivalent
Pre-operation, mean±SD, D	4.91 ± 3.77	−1.28 ± 0.94	−5.44 ± 3.83
Baseline, D	−0.29 ± 0.75	−0.49 ± 0.35	−0.53 ± 0.71
Post-treatment, D	−0.13 ± 0.67	−0.47 ± 0.38	−0.36 ± 0.64

**SD** standard deviation**, D** diopter.

**Table 3:** Comparisons between the subjects by the response to Alphagan-P treatment.

	Responders	Non- Responders	p
Number	30	31	
Age	38.1 ± 9.1	32.3 ± 10.3	**0.03**
Gender, male	43.3%	67.7%	**0.07**
Follow-up, months	7.6 ± 6.1	8.1 ± 8.0	0.79
Time for surgery to treatment, months	4.06 ± 4.0	5.0 ± 6.8	0.52
Pre-operation SE, mean±SD, D	−6.0 ± 3.1	−4.8 ± 4.3	0.22
Baseline SE, D	−0.82 ± 0.65	−0.26 ± 0.66	<**0.01**
Post-treatment SE, D	−0.31 ± 0.59	−0.41 ± 0.70	0.53
The difference in SE due to treatment, D	0.50 ± 0.50	−0.16 ± 0.26	<**0.01**
Baseline Cylinder, D	−0.55 ± 0.38	−0.42 ± 0.32	0.16
Baseline BCVA, logMAR	0.08 ± 0.1	0.05 ± 0.07	0.18
Post-treatment BCVA, logMAR	0.05 ± 0.06	0.03 ± 0.06	0.40
The difference in BCVA due to treatment, logMAR	0.15 ± 0.26	−0.003 ± 0.12	<**0.01**
Baseline UCVA, logMAR	0.36 ± 0.25	0.19 ± 0.17	<**0.01**
Post-treatment UCVA, logMAR	0.19 ± 0.17	0.19 ± 0.19	0.92
The difference in UCVA due to treatment, logMAR	0.14 ± 0.2	−0.003 ± 0.12	**0.01**

SE spherical equivalent, SD standard deviation, D diopter, BCVA best-corrected visual acuity, UCVA uncorrected distance visual acuity.

Multiple binary logistic regression confirmed these predictors for treatment response: UCVA (OR=70.6, *P* = .006), larger SE (OR=3.8, *P* = .004), and older age (OR=1.06, *P* = .03). Adverse effects (burning or stinging sensation and local allergic reaction) that led to treatment discontinuation occurred in 2 subjects (1.1%).

## Discussion

This is the first study to investigate using Brimonidine tartrate 0.15% ophthalmic solution (Alphagan-P) drops to treat post-LASIK residual myopia. After administrating this treatment, there was a significant improvement in the sphere and SE. Patients who responded to treatment were older, had higher baseline SE, and were female. Multiple binary logistic regression showed that the most significant predictors for response were lower baseline UCVA, larger SE, and older age. This might be described by the term “floor” effect: the greater potential for patients with lower baseline VA to acquire vision [16].

Our subject group’s age was 35 years, and it was nearing presbyopic age. In older patients we don’t plan for overcorrection so the chance of residual myopia, in this group of patients, might be higher. In addition, the demands or surgery expectations of older patients might be higher.

Previous studies comparing the effect of eye drops on residual myopia following refractive surgery are presented in Table 1. To our knowledge, all previously published data studied the effect of timolol eye drops exclusively. Reduced IOP causes less steepening of both sides of the cornea and lowers myopic refraction [16]. In addition, Shojaei et al. showed in a prospective randomized clinical trial that the SE improvement in patients receiving Timolol 0.5% eye drops lasted for at least six months after drops discontinuation [8].

As discussed by Kamiya et al., there is a forward shift of the cornea, which is not ectasia, in patients after refractive surgery, leading to a myopic shift that stabilizes six months post-operatively [17]. The effect of Alphagan-P for treating myopic shift following refractive laser surgeries could be explained by several mechanisms (Fig 1). First Alphagan-P treatment has an IOP-lowering effect which leads to rearward displacement of the cornea and flattening of its curvature [18]. Moreover, treatment with Alphagan-P leads to a pinhole effect through miosis, which promotes depth of focus. Shemesh et al. found significant reduction in pupil diameter among 90% of patients 6 hours after instillation of Alphagan-P under dark luminance [19].

This results in halo reductions, improved night vision and contrast, and reduced discomfort and subconjunctival hemorrhage size [13,20–22]. Previous` studies suggested that this effect was mainly limited to night vision, which can improve driving at night or working in dim illumination work environments Furthermore, the administration of Alphagan-P, through its miotic effect [11,23], shallows the AC and promotes forward displacement of the lens and the ciliary body, causing a hyperopic shift, subsequently reducing the myopic effect following refractive surgery.

Lowering the IOP may change and influence the refraction, hence Alphagan-P was argued to improve patients’ refraction. David et al. stated the significant correlation between myopia and high IOP early in 1985 [24]. Later on, IOP was found changing the corneal curvature as was discussed by Niemczyk et al., explained by the alter in the collagen density and orientation caused by the changes in the IOP [25]. Gunvant et al. also studied the influence of IOP over the corneal curvature, and found that for an increase of 1 mmHg of IOP there was a rise in mean corneal curvature of 0.84 mm [26]. In 2018, as further described by Mazzeo TJMM et al., reducing the IOP results in thinner corneal thickness and in reduced corneal K in over 2 diopters, which may improve the refraction significantly [27].

Our study reported that in 11% of patients, loss of 1 line was spotted. This can be attributed to ocular surface disorders caused by the Alphagan-P treatment. In Such cases, substantial lubrication treatment is recommended, and Alphagan-P treatment cessation might be considered.

The limitations of our study are its retrospective design, lack of a control group, and lack of examination of the rebound effects once treatment is stopped. If the refractive effects are solely determined by the degree of IOP decrease, patients may need to take IOP reduction medications constantly, with projected local adverse effects.

In conclusion, we demonstrated that topical treatment of Alphagan-P eye drops might be beneficial for correcting low residual myopia, where ablation retreatment may not be a valid option.
